# Virus classification – where do you draw the line?

**DOI:** 10.1007/s00705-018-3938-z

**Published:** 2018-07-24

**Authors:** Peter Simmonds, Pakorn Aiewsakun

**Affiliations:** 1 Nuffield Department of Medicine, University of Oxford, Peter Medawar Building, South Parks Road, Oxford, OX1 3SY UK; 2 Department of Microbiology, Faculty of Science, Mahidol University, Bangkok, 10400 Thailand

## Abstract

High-throughput sequencing (HTS) and its use in recovering and assembling novel virus sequences from environmental, human clinical, veterinary and plant samples has unearthed a vast new catalogue of viruses. Their classification, known by their sequences alone, sets a major challenge to traditional virus taxonomy, especially at the family and species levels, which have been historically based largely on descriptive taxon definitions. These typically entail some knowledge of their phenotypic properties, including replication strategies, virion structure and clinical and epidemiological features, such as host range, geographical distribution and disease outcomes. Little to no information on these attributes is available, however, for viruses identified in metagenomic datasets. If such viruses are to be included in virus taxonomy, their assignments will have to be guided largely or entirely by metrics of genetic relatedness. The immediate problem here is that the International Committee on Taxonomy of Viruses (ICTV), an organisation that authorises the taxonomic classification of viruses, provides little or no guidance on how similar or how divergent viruses must be in order to be considered members of new species or new families. We have recently developed a method for scoring genomic (dis)similarity between viruses (Genome Relationships Applied to Virus Taxonomy – GRAViTy) among the eukaryotic and prokaryotic viruses currently classified by the ICTV. At the family and genus levels, we found large-scale consistency between genetic relationships and their taxonomic assignments for eukaryotic viruses of all genome configurations and genome sizes. Family assignments of prokaryotic viruses have, however, been made at a quite different genetic level, and groupings currently classified as sub-families are a much better match to the eukaryotic virus family level. These findings support the ongoing reorganisation of bacteriophage taxonomy by the ICTV Phage Study Group. A rapid and objective means to explore metagenomic viral diversity and make evidence-based assignments for such viruses at each taxonomic layer is essential. Analysis of sequences by GRAViTy provides evidence that family (and genus) assignments of currently classified viruses are largely underpinned by genomic relatedness, and these features could serve as a guide towards an evidence-based classification of metagenomic viruses in the future.

## The diversity and classification of viruses

Virus taxonomy is an essential element in the description of viruses and acts as a unified catalogue of their vast diversity and genetic interrelationships. Viruses are assigned in a hierarchy of taxonomic levels by the International Committee on Taxonomy of Viruses (ICTV; https://talk.ictvonline.org/). Viral diversity is, however, far greater than that of other organisms, with major differences in their genetic material (RNA or DNA) and configurations (double or single stranded), as well as the orientation of their encoded genes. Viral genomes may, furthermore, be distributed across several segments, sometimes packaged together in a virion, or often in separate virus particles, all of which are needed to infect a cell for replication to occur. Viral genomes come in various sizes, reflecting their diverse replication mechanisms and cellular interactions, as well as the varying structural complexity of their virions. The smallest virus genomes range from less than 2,000 bases, containing two genes, to 2.5 million base pairs, containing over 2,500 genes [1]. Similarly, there is extraordinary variability in virus particle morphology and size; some virus particles show icosahedral or more complex symmetry, while others may form filamentous, rectangular, bullet, or even bottle-shaped nucleocapsids. Viruses infecting bacteria typically possess “tails” and “spikes”, structural complexes that attach to and pierce the otherwise impermeable bacterial cell wall and injects viral DNA into the cytoplasm.

The taxonomy of cellular organisms, including microorganisms such as bacteria and unicellular fungi, is built on a common evolutionary framework – ultimately all eukaryotes share a last common ancestor, distinct from those of bacteria and archaea representing the other domains of life. These deeper relationships are largely recoverable from their genetic relationships; the phylogeny of core genes, such as those for ribosomal proteins, provides a reasonable representation of their evolutionary origin and divergence many billions of years ago. Unfortunately, the diversity of viruses prevents such a reconstruction of virus evolutionary histories – they lack any equivalent set of universally conserved genes on which to construct a phylogeny [9, 11, 19]. Viruses appear to have appeared on several occasions as parasitic companions of the various prokaryotic and eukaryotic life forms that they infect [13].

The current taxonomy of viruses has itself gradually evolved since the formation of what is now the ICTV in 1966. The vastly and rapidly expanding knowledge of virus diversity since that time and the advent of molecular methods for virus discovery and genetic characterisation have greatly increased the number of taxa assigned, with the current totals being 9 orders, 131 families, 46 subfamilies, 803 genera and 4,853 species, following the ratification vote after the 49^th^ ICTV Executive Committee meeting [18]. Incorporating the hugely diverse collection of evolutionarily related (and perhaps unrelated) groups of viruses into a single, overarching framework represents a considerable and ongoing organisational achievement, and the resources and descriptive catalogues of viruses (9^th^ and 10^th^ Report - https://talk.ictvonline.org/ictv-reports/) are vital resources underpinning the whole virus classification field.

Nevertheless, you might discover on closer inspection that the unified virus taxonomy is a rather ramshackle construction, with taxonomic assignment rules often being based on quite different and inconsistent criteria between virus groups. For example, the assignment of viruses to the order *Herpesvirales* is based on their morphology, and is independent of genomic relationships, which do not place their members into a coherent genetic group. Contrastingly, membership of the order *Tymovirales* is based upon possession of genetically related RNA polymerase genes, irrespective of the highly variable virion structures of their members.

## Species-level classification

The species definition is particularly variable between virus groups. This taxonomic level was originally used as the primary division of viruses showing distinct disease patterns, epidemiology and host range. *Yellow fever virus*, *Carnation mottle virus* and *Salmon pancreas disease virus* are typical examples of literally hundreds of such disease-focussed designations. As nucleotide sequence information on classified species gradually accrued in the 1980s-1990s, it became clear that species were typically genetically distinct from each other, but there was no fixed sequence divergence threshold that defined members of the same and different species. As an example of what typifies large numbers of other species assignments throughout the ICTV taxonomy, members of different flavivirus species show pairwise distances in the RNA-dependent RNA polymerase (RdRp) gene of only 1.5% (between the species *Israel turkey meningoencephalomyelitis virus* and *Bagaza virus*), while members of *San Perlita virus* and *Ilheus virus* differ by 44%. As an example of even greater discrepancy, members of the species *Louping ill virus,* which infect grouse and sheep in upland Britain, lie entirely within the clade of the separate species *Tick-borne encephalitis virus*. The two were defined as separate species based on their marked differences in geographical range, host associations, and pathogenicity, and clearly not their genetic relationships in this case.

It has been entirely possible to maintain what is essentially a phenotypically based system of species assignments for many decades – these assignments do, after all, divide viruses into groups that differ in important medical, veterinary and agricultural properties that serve a major purpose for classification. Flexibility in what defines species is implicit in the polythetic species definition [27, 28] developed by Marc Van Regenmortel, in which constellations of properties, none of which would be individually essential for species inclusion or exclusion, are formulated to produce a highly intuitive and effective descriptive definition. Polythetic species definitions, indeed, have provided the basis for ICTV taxonomy assignments for nearly two decades. The difficulty that has arisen from this approach is that, increasingly, viruses are discovered by molecular methods and, most recently, by deep sequencing of environmental and other samples using high-throughput sequencing (HTS) methods. These techniques often simply provide a virus sequence but none of the typical sets of properties that might constitute a polythetic definition.

As HTS methods become increasingly used, and the catalogue of viruses known only by their sequence data continues to expand almost exponentially, genomics-based species assignments are clearly required to accommodate these into the ICTV taxonomy [10, 12, 21, 22, 31]. The difficulty is how to assign what often are purely sequence-based species thresholds in a manner that is not purely arbitrary and which is consistent with the taxonomy of other viruses whose phenotypic properties are known. Many opinions and proposals have been put forward on this crucial procedural issue over many years [7, 14, 29, 30], recently reviewed in reference [26]. However, in practical terms, all of these boil down to a central and seemingly irreconcilable dilemma – you can’t use descriptive species definitions for viruses where there is nothing to describe except its nucleotide sequence. On the other hand, disallowing sequence-only assignments produces an ICTV taxonomy that fails in its function as a proper catalogue of viral diversity. Sequence-only viruses are still viruses. It is simply that information on their properties has not been collected (yet) – hardly a reason for their permanent exclusion. We suggest that those involved in previous discussions and those expressing new views on the subject take the time to discuss future species definitions for viruses, to critically evaluate the various biological species definitions currently in use and to decide which concepts are most suitable for viruses in the future – the polythetic species is, after all, just one of the over 20-30 species definitions used or proposed in wider biology.

Virologists should not be daunted by the scale of the task ahead in making species assignment to the vast number of viruses identified by deep-sequencing methods. Recent descriptions of potentially tens or hundreds of new species, genus and family assignments for viruses isolated from arthropod and lower vertebrate host taxa [20, 23, 24] are really no different taxonomically from, and are indeed simpler in practical terms than classification tasks elsewhere in biology. A (possibly extreme) example is the still ongoing cataloguing of beetle species, with more than 400,000 species already assigned, each bearing a binomial, Latinised name and perhaps more than half a million to go.

## Family-level classification

While the species level of taxonomy is one that best describes what we would consider individual viruses in a broad sense, the family level is the fundamental taxonomic level by which viruses of different kinds are organised. Eukaryotic viruses are currently assigned into a total of 111 families, with distinct genome configurations, virion morphologies, replication strategies and host interactions. Many of the groupings first described in early ICTV reports, generally based on electron microscopy appearance and genomic material, have stood the test of time – herpesviruses, poxviruses and rhabdoviruses are instantly and recognisably distinct morphologically, and their separateness has been verified by detailed analysis of their genome structures and replication mechanisms.

The ICTV taxonomy, however, provides little information that might guide decisions on what is needed to justify the creation of new virus families or orders. The ICTV Code provides this as a definition of a virus family:

*A family is a group of genera (whether or not these are organized into subfamilies) sharing certain common characters*

building on:

*A genus is a group of species sharing certain common characters*

which, frankly, is equally uninformative. Although new families have continued to be assigned in the last three decades, there is no real systematic information or guidance on what virological, structural or genomic attributes would support the family-level assignments that have been made, and what may be used in the future. For example, family members must presumably share homologous genes, but how many and how similar? Must they have related genome organisations? Should they look similar by electron microscopy? Most crucially in this “metagenomic” era, are there any family-defining attributes recoverable from a virus sequence alone – or is it essential to visualise particle morphology and to determine some of the physical attributes of the virus, such as host range, cell tropism and pathogenicity, as part of an extended family descriptive definition? The ICTV offers no guidance. Perhaps as a direct result of this uncertainty, picorna-like, flavi-like, circo-like and a vast range of other (family)–like virus descriptions are to be found in literally hundreds of papers indexed by PubMed. In many cases, it is as if their authors, too, lacked the information or confidence to assign new families for newly discovered viruses. Guidance on this issue and, ideally, a better-defined set of criteria for family assignments seems long overdue – this is indeed required if the ICTV is going to embrace the growing dataset of virus genome sequences from metagenomic datasets.

## Development of GRAViTy

It was with this background that we set out to examine whether there were any consistent genomic attributes of eukaryotic virus families that correlated with their current family-level ICTV taxonomy relationships [5]. Investigating this involved extracting and analysing a wide range of genetic metrics from the set of currently classified viruses, including organisational features (gene complements and gene orders), possession of homologous genes and their amino acid sequence identity. These features were then systematically evaluated for their ability to recover the taxonomy of all currently classified eukaryotic viruses in the ICTV Master Species List [5]. The logic of this exercise was based on the premise that genome features that were informative and predictive of taxonomic relationships could then be extracted from currently unassigned viruses from their sequences alone (including the vast number of “-like” viruses), and informed decisions on their assignments could be made. While this represents an entirely bioinformatic approach, it ensures that any family or other-level assignments of sequence-only viruses are broadly consistent with existing taxonomic placements of viruses classified by other means.

This method, “Genome Relationships Applied to Virus Taxonomy” (GRAViTy), was applied to the complete list of 3,854 classified eukaryotic viruses with complete genome sequence data. Information such as gene contents, orientations and protein profiles were extracted from each genome sequence and systematically compared through the calculation of a composite generalised Jaccard (CGJ) distance, a metric that captures and weights the contributions of different genome features between pairs of viruses. This use of multiple features extracted from viral sequences contrasts with traditional phylogenetic methods, in which virus relationships are often calculated from small, highly conserved portions of their genomes, such as the catalytic core of the RdRp gene.

**Fig. 1:**
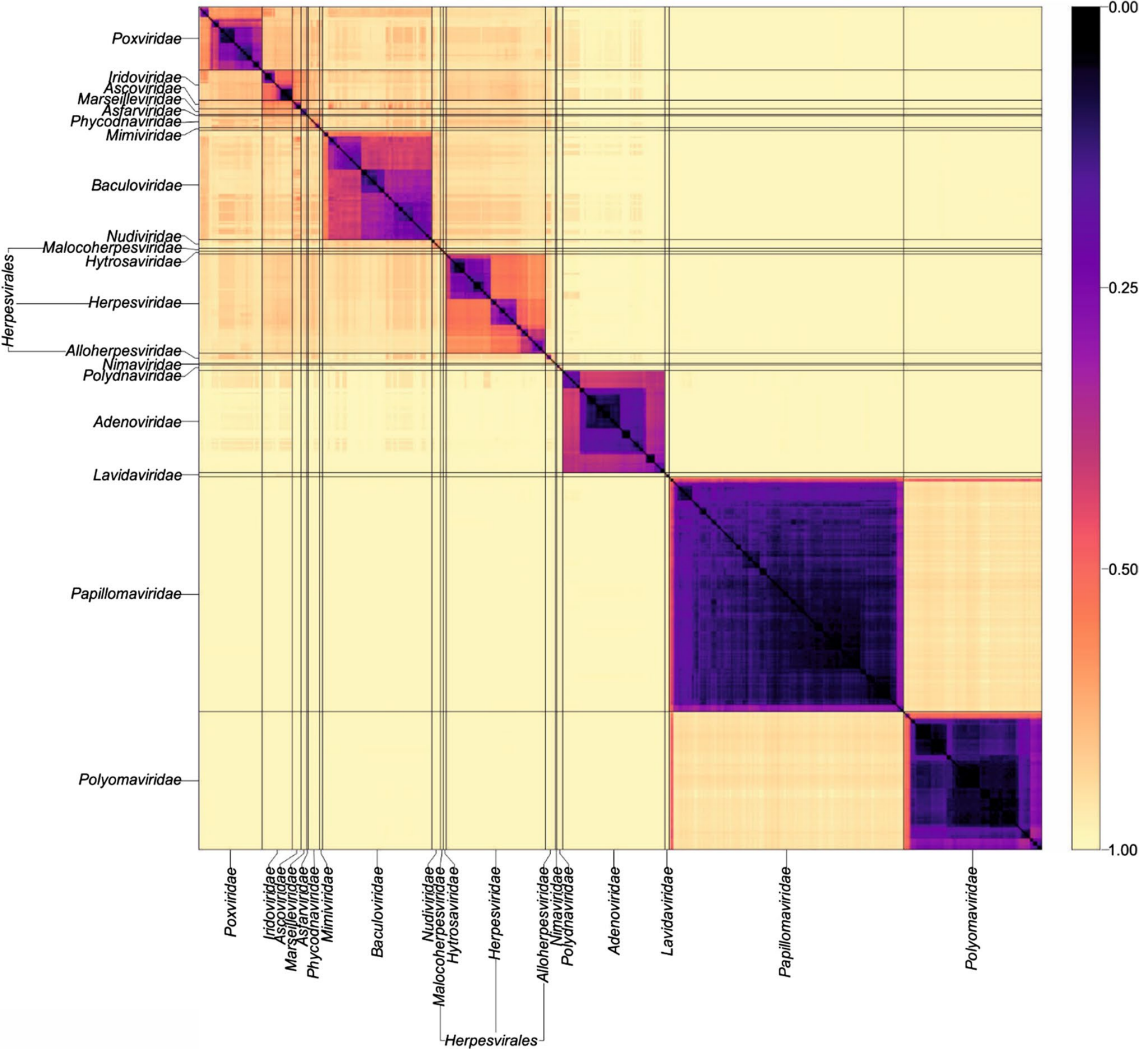
Heat maps of CGJ distances between dsDNA. Pairwise CGJ distances were computed between each sequence plotted as a heat map using colour-coded points (see scale on the left of the figure). The light grey solid lines indicate boundaries between each virus taxonomic group. Annotations for each virus family and order are shown on the axes. This figure has been reproduced from reference [5]

**Fig. 2:**
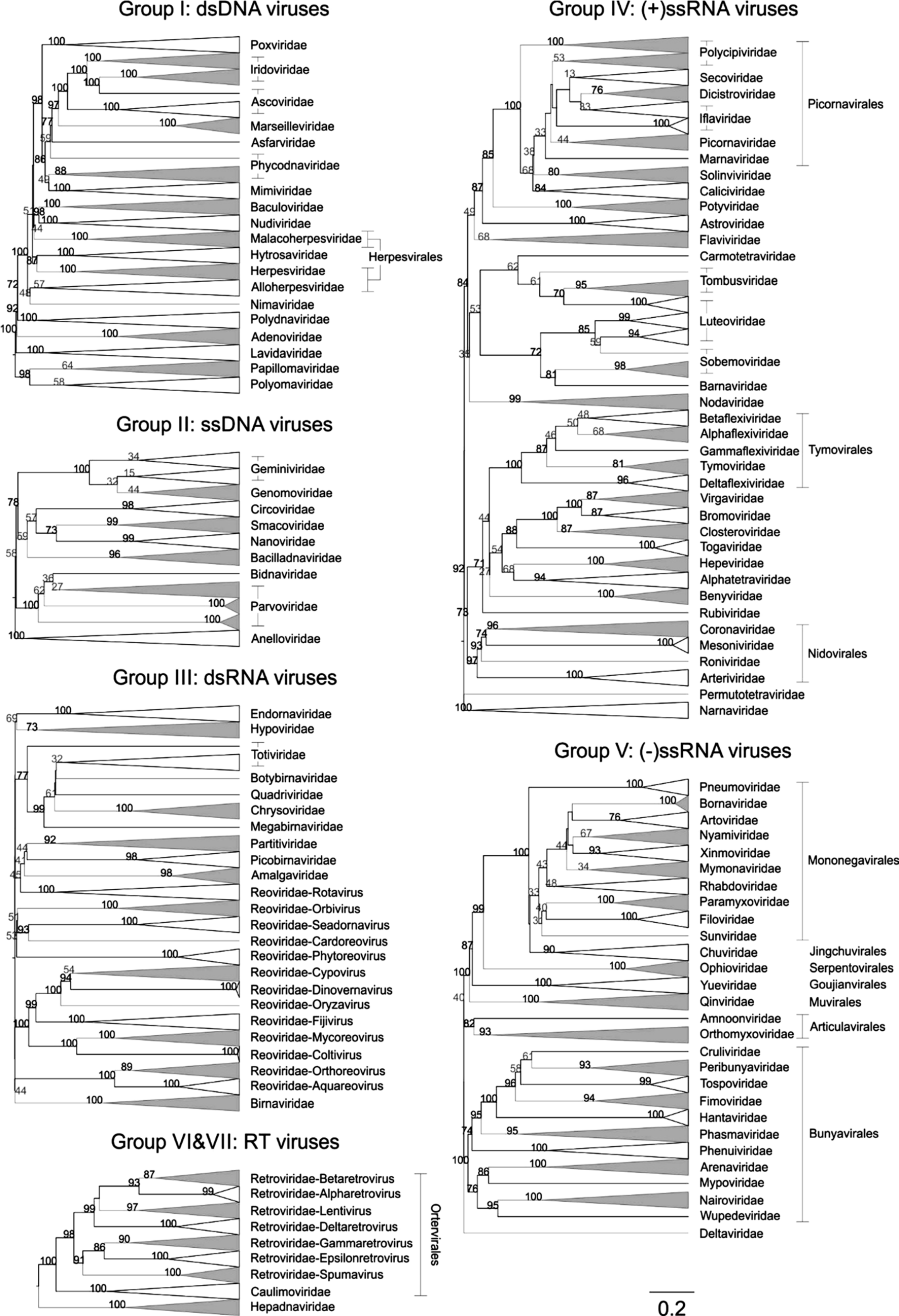
Virus dendrograms based on CGJ distances. UPGMA dendrograms were constructed from pairwise distance matrices, and the tips are labelled with current ICTV family and genus assignments. Virus taxonomy at the order level is also shown to the right of the dendrograms. The scale bar for the CGJ distance is shown at the bottom. Bootstrap clade support values of ≥ 30% are shown on the branches. Values ≥ 70% are in black, otherwise are in grey. Bootstrap support values for collapsed clades are shown regardless of the values. This figure has been modified from Fig. 2 in reference [5]

Pairwise distances between members of particular virus groups, such as dsDNA viruses (Baltimore group I), for example, can be visualised as a heat map (Fig. 1) or as dendrograms (Fig. 2). The highly encouraging finding from this analysis was the close concordance between sequence groupings and their current ICTV assignments into families for viruses of all configurations (dsDNA, ssDNA, dsRNA, (+)ssRNA, (-)ssRNA and retro-transcribing viruses). GRAViTy showed 95.7%-100% (mean, 99.1%) sensitivity and 99.3%-100% (mean, 99.8%) specificity for the virus assignments with the current virus taxonomy. These associations are all the more striking for being derived from genome features without pre-selection for which ones might be considered to be informative, and furthermore being generated without having to construct multiple sequence alignments with its attendant but often hidden assumptions and problems. The analysis revealed a primary division of viruses at the family level that, with very few exceptions, was readily identifiable as tight clusters with ≥70% bootstrap support in the dendrogram (Fig. 2). The small number of exceptions in other Baltimore groups were in themselves often highly informative – the separation of rubella virus (genus *Rubivirus*) from the rest of the family *Togaviridae* concurs with a variety of other evidence that it should be re-classified into a separate family. Some virus families turned out to be far more diverse and often polyphyletic, including *Rhabdoviridae* and *Reoviridae,* and these represent future candidates for reorganisation of their taxonomy.

With this newly developed ability to predict family memberships from genomic sequences alone, we next analysed large datasets of virus sequences derived from metagenomic datasets to determine the extent to which they might be classified as new potential families, or as members of existing ones. Using the same metrics that differentiated virus families in each Baltimore group, we were able to provisionally assign viruses in this combined metagenomic dataset as four new families of ssDNA viruses, four dsRNA virus families, potentially as many as 101 new family-level groupings of (+)ssRNA viruses and 16 new (-)ssRNA families. Clearly, these results are preliminary, and conclusions about such a large number of new families require corroboration with other methods, including RdRp phylogenies in the case of future RNA viruses, as is the extension of classification to genus and species levels. What we can say, however, is that future assignments will certainly approximate to relationships that GRAViTy has shown. Furthermore, the new frameworks for each Baltimore group depict sets of virus relationships that are consistent with and extend virus taxonomy in a manner that effectively follows the rules used in the current classification.

## Bacterial and archaeal virus (phage) taxonomy

Viruses infecting bacteria and archaea are extraordinarily abundant and diverse. Almost all of the currently classified bacterial viruses are, however, assigned to just three families of tailed phages, namely the *Myoviridae*, *Podoviridae* and *Siphoviridae* [2], a division based upon their distinct morphologies (myo-: long contractile, sipho-: long non-contractile and podo-: short tails). While it has been appreciated for many years that members of these families are highly diverse genetically, there has been little to no attempt to match these family assignment categories to those used for eukaryotic viruses. There is, consequently, no real idea of the extent to which bacteriophage assignments are taxonomically equivalent or not. This may not have mattered too much when different and largely independent scientific communities were engaged in their separate phage and eukaryotic virus research programmes, but the advent of metagenomic virus sequence data challenges this division. Bacterial, archaeal, and eukaryotic viruses abound in environmental samples that typically generate the largest metagenomic datasets – how can there be different rules for their classification when their hosts are not necessarily known?

**Fig. 3:**
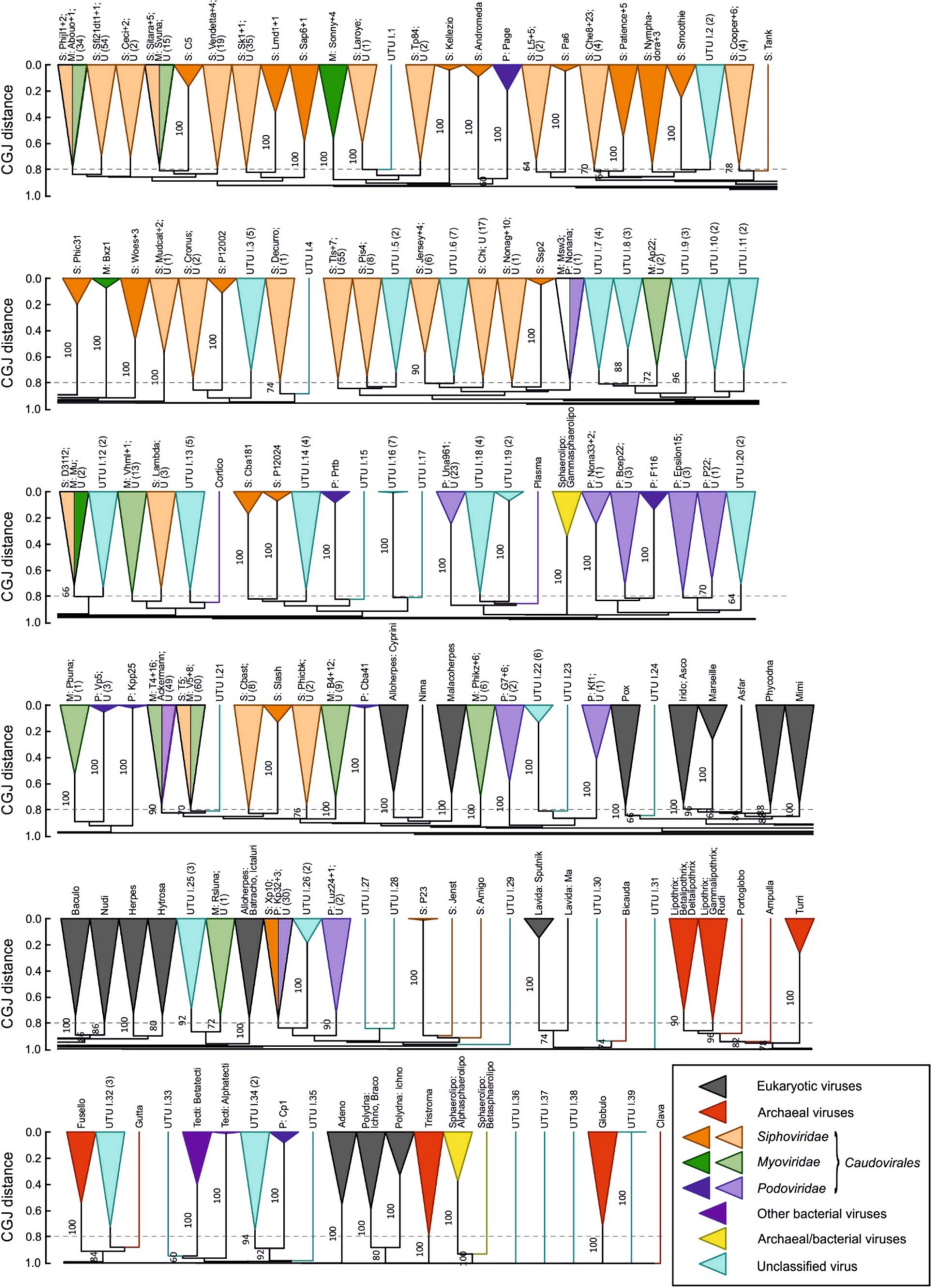
UPGMA dendrogram of classified and unclassified dsDNA viruses (Baltimore group I) based on CGJ distances. The dendrogram is divided into six separate lines to represent the 139 clades present in the dataset. Tips are labelled with genus for members of the *Caudovirales*; abbreviated as S: *Siphoviridae*, M: *Myoviridae*, and P: *Podoviridae*, and by family/genus for other bacterial, eukaryotic and archaeal viruses, or by accession number codes for unclassified viruses. The scale bar for CGJ distance is shown at the left of each line, and the 0.8 threshold that corresponds to eukaryotic family groupings is shown as a grey dotted line. Bootstrap re-sampling was performed with pruned signature tables as described previously [5]. Clades were coloured based on host origin according to the key; those containing both classified and unclassified (U) sequences are shown in a lighter shade. The 39 new candidate unassigned taxonomic units (UTUs) arising from the inclusion of current unclassified viruses are shaded in light blue. This figure has been reproduced from reference [4]

We addressed this question directly through analysis of sequence datasets by GRAViTy that incorporated both eukaryotic and prokaryotic viruses in Baltimore groups I-IV (dsDNA, ssDNA, dsRNA and (+)ssRNA) [4]. This analysis revealed quite different sequence relationships, particularly between those of members of the tailed phage families *Siphoviridae, Myoviridae* and *Podoviridae* (assigned to the order *Caudovirales*) and those of eukaryotic viruses. Members of the same phage family were generally far more divergent from each other and, despite their morphological similarities, frequently possessed genes that showed no detectable homology with each other (Fig. 3). If we were to apply the same metric of genetic relatedness observed between eukaryotic virus families, we might split the *Caudovirales* into as many as 70 family-level groupings and a further 37 groups comprising currently unclassified bacteriophage sequences available in public databases. These totals are, nevertheless, far more consistent with their own genetic diversity and that of the vast range of bacterial hosts they infect.

**Fig. 4:**
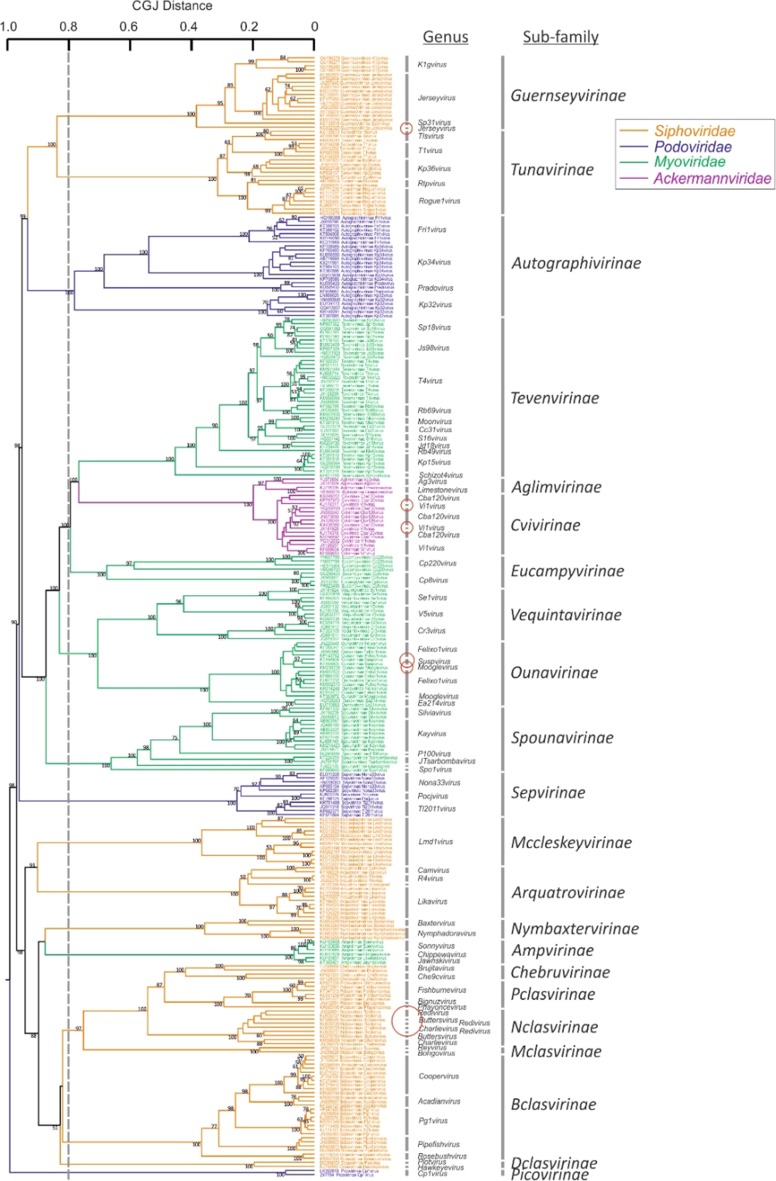
Dendrogram of members of the order *Caudovirales* that have subfamily assignments. Taxa are colour coded for family (see key). Minor discrepancies between genus assignments and phylogeny are shown in red circles. This figure has been reproduced from reference [4]

Examining sequence relationships a little further, it was apparent that the subfamily-level groupings among tailed phages were generally more consistent with family assignments of eukaryotic viruses where these have been made (Fig. 4). These findings fully support ongoing reclassifications by the ICTV of subfamilies, such as *Spounavirinae* and *Vi1virus* taxa, as new virus families based on genome relationships rather than phonotypic properties, such as virion appearance and presumed host [6, 15, 16]. The traditional morphology-based classifications of prokaryotic viruses are, indeed, increasingly untenable even in principle, since the bulk of new phage sequences to be classified derive from metagenomic datasets, where few or no phenotypic attributes are available.

## Concluding remarks

The ICTV is committed to the incorporation of viruses known only by their genome sequence into the current taxonomy [3, 25]. This is a logical step if the ITCV taxonomy is to remain relevant as a guide to viral diversity; viral sequences reconstructed from HTS data represent viruses as much as those whose phenotypic properties are known (at least in part). However, as reviewed previously [26], classifications built on sequences alone directly challenge many of the concepts and assumptions of polythetic and other descriptive definitions of virus species.

They also reveal gaping holes in how the various taxonomic levels are defined – the ICTV code provides no indication of how different viruses have to be, either in terms of their phenotypic properties or in their genetic relatedness, to be considered members of the same or different species, genus or family. Given the importance of the family level in virus taxonomy, it is remarkable that there is no information about what features might define a family and enable new families to be assigned in a consistent way. The application of bioinformatic programs like GRAViTy, vContact and others [4, 5, 8, 17] will be important in documenting those genomic features that underlie this and other taxonomic levels of currently classified eukaryotic and prokaryotic viruses. Such approaches are essential if future sequence-only assignments are to be made from a strong evidence base. While we can all foresee a future with a vastly expanded taxonomy of viruses, we think we should also preserve and respect as much of the current taxonomy as we can – the delineation of how current taxonomy works at a genomic level is an important initial step in this process.
